# *Wohlfahrtiimonas chitiniclastica* Bacteremia Hospitalized Homeless Man with Squamous Cell Carcinoma

**DOI:** 10.3201/eid2409.170080

**Published:** 2018-09

**Authors:** Yuichi Katanami, Satoshi Kutsuna, Maki Nagashima, Saho Takaya, Kei Yamamoto, Nozomi Takeshita, Kayoko Hayakawa, Yasuyuki Kato, Shuzo Kanagawa, Norio Ohmagari

**Affiliations:** National Center for Global Health and Medicine, Tokyo, Japan

**Keywords:** bacterial infections, bacteremia, bacteria, homeless persons, carcinoma, wound infection, mass spectrometry, matrix-assisted laser/desorption-ionization time-of-flight, MALDI-TOF, RNA, ribosomal, 16S, Japan, *Wohlfahrtiimonas chitiniclastica*

## Abstract

We report a case of *Wohlfahrtiimonas chitiniclastica* bacteremia in an elderly man in Japan who had squamous cell carcinoma. Blood cultures were initially negative for *W. chitiniclastica* but were positive on day 20. Careful attention needs to be paid to this organism in patients who have chronic wounds with maggots.

We report *Wohlfahrtiimonas chitiniclastica* bacteremia in a 75-year-old man in Japan who had squamous cell carcinoma on his shoulder. In September 2016, an unidentified patient was found unconscious on the ground by a passerby and admitted to the emergency department of the National Center for Global Health and Medicine (Tokyo, Japan). He had a necrotic lesion on his left shoulder with maggots. Blood analysis showed leukocytosis (26.61 × 10^9^ cells/L [reference range 3.30–8.60 × 10^9^ cells /L]), thrombocytosis (626 × 10^9^/L [reference range 158–348 × 10^9^/L]), anemia (hemoglobin, 9.6 g/dL [reference range 13.7–6.8 g/dL]), and elevated C-reactive protein (87.9 mg/L [reference range 0.00–1.40 mg/L]). Albumin was 2.4 g/dL (reference range 4.1–5.1 g/dL) and calcium was 12.6 mg/dL (reference range 8.8–10.1 mg/dL). He was diagnosed with disturbance of consciousness caused by hypercalcemia and was hospitalized.

After saline infusion and intravenous cefazolin (3 g/d) were initiated, the patient’s condition improved. A blood culture taken at the time of admission grew *Peptoniphilus harei*. A swab culture of the ulcer site grew *Proteus mirabilis*, *Morganella morganii*, and *Kerstersia gyiorum*. A biopsy was performed on day 3, and the patient was diagnosed with squamous cell carcinoma. Enhanced computed tomography scanning revealed an ulcer and ring-enhancing lesion on his left shoulder (which was suspected of being a tumor or abscess) and multiple enlarged lymph nodes and 10-mm pulmonary nodules in the right lung.

On day 20, the patient had fever and disturbance of consciousness; therefore, he was transferred to the Infectious Disease department of the hospital. Intravenous therapy with vancomycin (1.5 g/d), cefepime (3 g/d), and metronidazole (1,500 mg/d) was initiated, and the patient’s fever and consciousness improved. Two cultures of blood taken on day 20 grew *P. mirabilis*, *M. morganii*, *Streptococcus anginosus*, *Streptococcus agalactiae*, *Bacteroides fragilis*, and gram-negative rods. After we obtained the culture results, vancomycin was stopped in accordance with the susceptibility test results. We identified the gram-negative rods as *W. chitiniclastica* by using matrix-assisted laser desorption/ionization time-of-flight mass spectrometry (Bruker Daltonics, Billerica, MA, USA), which showed scores of 2.239. We further confirmed the isolate to be *W. chitiniclastica* by using 16S rRNA sequencing; the isolate was 99.08% identical to strain S5 (GenBank accession no. AM397063). We assessed the isolate’s antimicrobial susceptibility profile (Table). The patient improved and was later discharged to another hospital.

**Table:** Antimicrobial susceptibility profile of *Wohlfahrtiimonas chitiniclastica* from a blood culture from a patient treated at the National Center for Global Health and Medicine, Tokyo, Japan, 2016.

Antimicrobial drug	MIC, mg/L susceptibility profile
Piperacillin	≤8, S
Piperacillin/tazobactam	≤8, S
Ceftazidime	≤4, S
Cefepime	≤2, S
Aztreonam	≤4, S
Imipenem/cilastatin	≤1, S
Meropenem	≤1, S
Amikacin	≤8, S
Gentamicin	≤2, S
Tobramycin	≤2, S
Minocycline	≤2, S
Levofloxacin	1, S
Ciprofloxacin	1, S
Trimethoprim/sulfamethoxazole	≤2, S

*S, susceptible.

*W. chitiniclastica* is a gram-negative, short, facultative anaerobic, straight-rod gammaproteobacterium that was first isolated from the parasitic fly *Wohlfahrtia magnifica* (*1*). This fly has not been reported in Japan. However, *W. chitiniclastica* has also been isolated from the *Chrysomya megacephala* fly, and this species has been reported in Japan (*2*), and from from the *Musca domestica* housefly, which is widely distributed all over the world (*3*). Campisi et al. reported that the *Lucilia sericata* fly might be a vector for *W. chitiniclastica* (*4*); this fly is common and widely distributed throughout Japan, and a case of cutaneous myiasis on skin cancer was reported (*5*). Unfortunately, we could not collect maggots from this patient because they were rapidly discarded at the emergency department.

Worldwide, few human cases of *W. chitiniclastica* infection have been documented. *W. chitiniclastica* has been described as a zoonotic pathogen (*6*) and reported from Hungary, Egypt, Niger, Germany, India, France, China, Argentina, Estonia, the United Kingdom, and the United States (*4**,**7**,**8*). Rebaudet et al. (*9*) described the first human case of bacteremia attributable to *W. chitiniclastica,* which occurred in a 60-year-old homeless woman from southeastern France who had a history of alcoholism. Other human cases of *W. chitiniclastica* bacteremia were reported from Argentina (*10*) and the United Kingdom (*4*). Recently, a bacteremia case in a 72-year-old man was reported from Hawaii, USA (*1*).

Risk factors for *W. chitiniclastica* infection are poor personal hygiene, alcoholism, peripheral vascular disease, and chronic open wound (*8*). The patient we describe had a chronic wound because of squamous cell carcinoma, and the associated maggots were thought to be the transmission route. At admission, blood and swab cultures grew polymicrobial isolates without *W. chitiniclastica,* as confirmed by using matrix-assisted laser desorption/ionization time-of-flight mass spectrometry. However, a blood culture on day 20 was positive for *W. chitiniclastica*. The patient probably was infected with *W. chitiniclastica* during hospitalization. Cases of *W. chitiniclastica* infection (with or without bacteremia) were reported as parts of polymicrobial infections (*1**,**4**,**7**,**8*). *W. chitiniclastica* might have first infected this patient’s ring-enhancing lesion as part of a polymicrobial infection. Because *W. chitiniclastica* was undetected in blood and swab cultures at admission, the organism might have entered the bloodstream during hospitalization.

This patient improved after intravenous therapy with cefepime and metronidazole. Previously reported *W. chitiniclastica* bacteremia cases were treated with combination antimicrobial therapies, including cefuroxime plus metronidazole plus clarithromycin (*4*), ceftazidime plus amikacin (*10*), piperacillin/tazobactam plus clindamycin plus vancomycin (*1*), ceftriaxone monotherapy (*9*), and meropenem monotherapy (*1*). Two of the 5 cases were fatal (*1*,*10*).

Clinicians should be attentive to the possibility of *W. chitiniclastica* infection in patients who have chronic wounds with maggots and poor hygiene. Clinical suspicion is warranted even if blood and swab cultures are initially negative for *W. chitiniclastica*.
